# Sleep-dependent enhancement of emotional memory in early childhood

**DOI:** 10.1038/s41598-018-30980-y

**Published:** 2018-08-22

**Authors:** Laura B. F. Kurdziel, Jessica Kent, Rebecca M. C. Spencer

**Affiliations:** 1 Department of Psychological & Brain Sciences, University of Massachusetts, Amherst, Massachusetts USA; 2 Neuroscience & Behavior Program, University of Massachusetts, Amherst, Massachusetts USA; 3 Department of Psychology, Merrimack College, North Andover, Massachusetts USA; 4 Commonwealth Honors College, University of Massachusetts, Amherst, Massachusetts USA

## Abstract

Naps in early childhood support declarative memory consolidation. However, emotional memories are unique in the neural basis of encoding as well as the sleep physiology underlying consolidation. Specifically, while consolidation of declarative memories has been associated with slow wave sleep, a prevailing theory suggests that REM sleep is necessary for consolidation of memories with emotional valence. Thus, we presented children (34–64 months) with faces paired with mean or nice descriptions. There were no significant main effects of emotional valence on recognition memory. Change in memory accuracy also did not differ when probed after a nap compared to the change in memory accuracy after an interval awake. However, when memory was probed again following overnight sleep, the change in memory accuracy was greater if the child napped the previous day. Greater nap slow wave activity was associated with greater memory decay during the nap. Yet nap slow wave activity also predicted greater overnight improvement in memory. These results suggest that sleep bouts can interact to benefit memory in early childhood.

## Introduction

Early childhood is a critical time for emotional development as language skills and the ability to regulate thoughts and behaviors emerge^1^. To capitalize on this developmental window, socio-emotional curriculum is now recommended in early education settings. Socio-emotional curriculum has been shown to increase prosocial behavior and improve long-term academic success. Thus, enhancing emotional learning at this young age has wide-reaching and long-lasting impact.

In adults, emotion processing and emotional memory are enhanced over sleep. Anecdotal evidence from caregivers and experimental evidence in toddlers suggests that naps support emotional regulation in early childhood. For example, children exhibit more frustration when faced with an unsolvable puzzle when nap deprived compared to rested. Moreover, emotional attention biases are reduced following a nap compared to an equal interval awake.

Yet, it is unknown whether emotionally valenced memories are also consolidated over naps in early development. Declarative memories have been shown to be consolidated over sleep in early childhood, thus naps may likewise benefit emotional memories. However, the REM Sleep Hypothesis of Emotional Processing holds that emotional memory consolidation requires REM sleep^2^ consistent with studies in adults^3–5^. Naps in early childhood lack this sleep stage^6^. Thus, naps may not be sufficient for emotional memory consolidation. If such were the case, REM in subsequent overnight sleep may support delayed emotional memory processing.

In spite of overwhelming support of REM’s role in emotional memory consolidation, recent studies suggest slow wave sleep (SWS) – specifically slow wave activity (SWA; spectral power in the delta band) in SWS – may also play a role. For example, selective memory for negative aspects of complex scenes is positively correlated with both SWS and SWA during a nap, although correlated with nocturnal REM sleep^7^. Likewise, negative memories selectively cued during SWS are better remembered than uncued items^8^. Such effects have been specifically associated with noradrenergic activity in SWS^8^. As such, emotional memories may benefit from SWS presence in the naps, which accounts for 42% of naps at this age.

**Figure 1:**
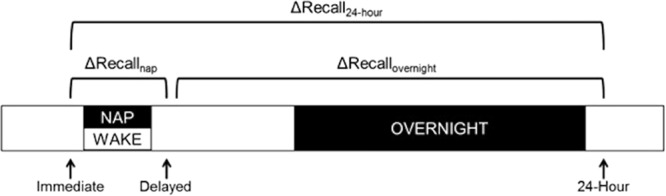
Encoding was followed by an immediate recognition memory probe. Recognition memory was probed again following a nap/wake interval (Delayed) and again the following morning (24-Hour).

To address whether naps contribute to memory consolidation for items with emotional valence in early childhood, we examined performance on an emotional memory task following a nap and an equivalent interval spent awake in young children using a within-subjects design. We also considered that REM in overnight sleep may provide a delayed benefit and, thus, assessed memory again the following day (Fig. 1). To assess memory for emotional information, we adopted a task used by Kinzler and Shutts^9^. This task was selected for three reasons: **(1)** it is age appropriate (as opposed to IAPS stimuli); **(2)** an emotional distinction is evident in behavior at this age (as evidenced by greater memory for negative compared to positive items at encoding^9^); and **(3)** enough items could be encoded to probe at three recall stages: baseline (immediately after encoding), following the nap/wake interval, and again the following day.

Polysomnography (PSG) was recorded in both nap and overnight sleep in a subset of participants to clarify the role of REM and SWS. We hypothesized that both positive and negative items would be remembered better following an interval with a nap relative to an equivalent interval spent awake. This hypothesis is based on: **(1)** children are observed to be both grumpy and giddy when nap-deprived suggesting naps may contribute to processing of both positive and negative emotions; and **(2)** naps reduce both positive and negative emotional attention biases in children at this age^10^. However, an alternative hypothesis is that negative memories would not benefit from the nap while positive memories would be greater following this mid-day sleep bout. Supporting this alternative: **(1)** REM sleep has been associated with negative memory consolidation^4,5^ and may not be involved in positive memory processing. Thus, the absence of REM from the nap would leave negative memories unchanged while positive memories, like neutral memories^6^, may benefit from non-REM sleep spindles; **(2)** a preliminary study in young adults suggested that sleep deprivation impairs positive and not negative memories^11^. As such, positive memories may be better remembered following a nap compared to wake (i.e., a nap benefit) while negative memories may be impervious to the effects of nap deprivation (the wake condition), particularly for children who nap habitually. Although^9^ neutral items were not included, consistent with Kinzler and Shutts^9^, this paradigm was sufficient to address these alternatives.

## Results

Of the 64 children (43 female; *M* = 51.55 months, *SD* = 7.23, range = 34–64 months) tested, 1 was unable to be wake promoted, 3 were unable to be nap promoted, and 3 were absent on one testing day, and were therefore excluded from analyses. In addition, as we would not expect sleep to yield performance benefits if the nap was of insufficient length, we further excluded children (n = 8) whose nap length was less than one standard deviation (*SD* = 22.99 min) below the mean (*M* = 71.92 min). This left a remaining sample of 49 children (30 female; n = 29 classroom, n = 20 laboratory; *M* = 51.55 months, *SD* = 7.16, range = 34–64.30 months) in the following analyses. Note that 2 children were absent for the 24-hr recognition phase but are included in all other analyses. Children tested in the laboratory compared to those tested in the classroom did not differ in age (*t*(47) = −0.688, *p* = 0.495), nap length (*t*(47) = 0.370, *p* = 0.713), or accuracy across any of the recognition phases (all *p*’s greater than 0.200); as such, behavioral data was collapsed to increase power.

Children’s self-report of both sleepiness and mood in session 1 (following immediate recognition) did not differ across conditions (*p*’s > 0.209). Experimenter ratings of mood also did not differ across conditions. However, experimenters did rate children as significantly more sleepy in the nap condition after both immediate recognition (*t*(18) = 2.348, *p* = 0.031; 95% CI: [0.039, 0.698]) and delayed recognition (*t*(19) = 3.943, *p* = 0.001; 95% CI: [0.281, 0.919]). It should be noted that average sleepiness was still rated as low in the nap condition for both the immediate (*M* = 1.45, *SD* = 0.60) and delayed recognition (*M* = 2.1, *SD* = 0.71) on the 5-item scale. Further, sleepiness levels at immediate recall (*r* = −0.072, *p* = 0.764) and delayed recognition (*r* = 0.286, *p* = 0.222) were not associated with memory performance across the nap bout, nor across the wake bout (immediate: *r* = 0.005, *p* = 0.983; delayed: *r* = 0.025, *p* = 0.915). Similarly, sleepiness at immediate recall was not associated with performance change across the 24-hr period in either the nap (*r* = −0.026, *p* = 0.913) or the wake condition (*r* = 0.047, *p* = 0.847).

### Memory Performance

Contrary to the findings of Kinzler and Shutts^9^, across conditions there were no significant differences between immediate recognition accuracy of negative and positive stimuli (*F*(1,48) = 0.029, *p* = 0.865). There was, however, a main effect of condition on immediate recognition accuracy (*F*(1,48) = 5.079, *p* = 0.029, ηp^2^ = 0.096) such that the baseline memory performance was greater prior to the wake condition (*M* = 72.4%, *SD* = 19.5%) compared to the nap condition (*M* = 63.7%, *SD* = 24.6%). For this reason, we focus on change in performance (as opposed to comparison of post-nap/post-wake performance; for absolute recognition memory, see Table 1).

**Table 1:** Absolute memory recognition accuracy scores for each session.

Session	Condition	Emotion	n	Mean	SD
Immediate Recall	Nap	Negative	49	62.92	31.525
	Wake	Negative	49	72.51	24.714
	Nap	Positive	49	64.39	29.132
	Wake	Positive	49	72.20	27.331
Delayed Recall	Nap	Negative	48	60.04	32.673
	Wake	Negative	49	67.71	28.031
	Nap	Positive	49	66.43	29.404
	Wake	Positive	49	67.10	26.747
24-Hr Recall	Nap	Negative	47	69.94	31.439
	Wake	Negative	46	60.17	36.823
	Nap	Positive	47	63.51	37.420
	Wake	Positive	46	59.09	34.979

**Figure 2:**
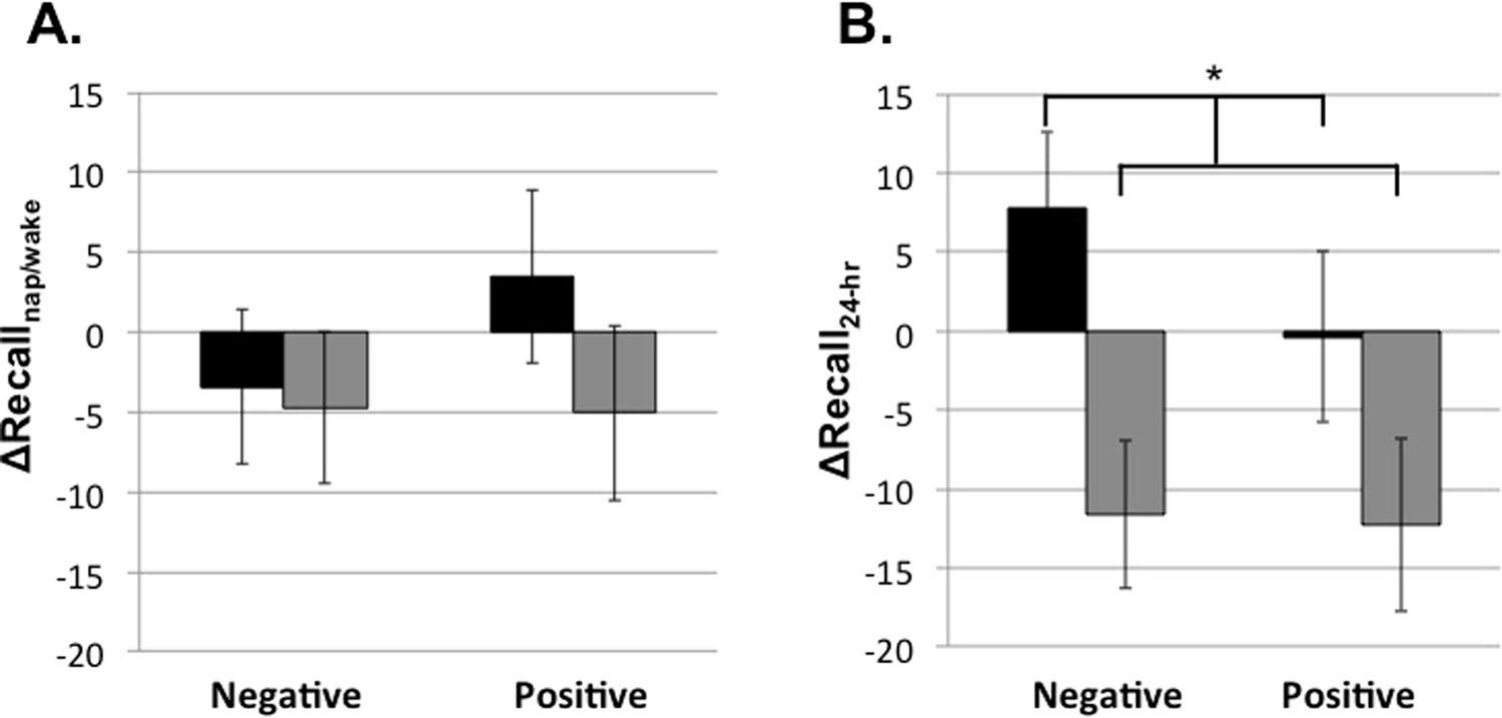
Nap-dependent benefits on emotional memory (change in % correct across the delay) were not observed initially (**A**) when comparing the nap condition (black bar) to the wake condition (grey bar). Effects were only observed following overnight sleep (**B**), reflecting a delayed benefit of the nap. Error bars represent SEM.

Counter to our prediction, performance changes over the nap did not differ from those over wake (ΔRecall_nap/wake_; *F*(1,47) = 0.678, *p* = 0.414; Fig. 2A). In addition, there was no main effect of emotion (*F*(1,47) = 0.285, *p* = 0.596), nor a significant interaction between emotion and condition (*F*(1,47) = 0.733, *p* = 0.396). However, when change in memory accuracy was assessed 24-hrs after learning (ΔRecall_24-hr_), memory recall was greater in the nap compared to the wake condition (main effect condition: *F*(1,43) = 4.523, *p* = 0.039; ηp^2^ = 0.095; Fig. 2B). The nap led to protected memory (one-sample t-test; *t*(46) = 0.758, *p* = 0.452; 95% CI: [−6.180, 13.650]), whereas wake resulted in significant forgetting (one-sample t-test; *t*(45) = −2.819, *p* = 0.007; 95% CI: [−20.590, −3.430]). Positive and negative stimuli were similarly benefited by the nap (main effect emotion: *F*(1,43) = 0.234, *p* = 0.631; emotion x condition: *F*(1,43) = 0.286, *p* = 0.595).

Given that changes in memory were observed across the 24-hr period, we considered whether overnight sleep alone was sufficient for these effects. Performance changes across the overnight sleep bout (ΔRecall_overnight_) were not significantly different across conditions (*F*(1,43) = 0.952, *p* = 0.335) nor emotion categories (*F*(1,43) = 1.387, *p* = 0.245; emotion x condition: *F*(1,43) = 0.606, *p* = 0.441).

**Figure 3:**
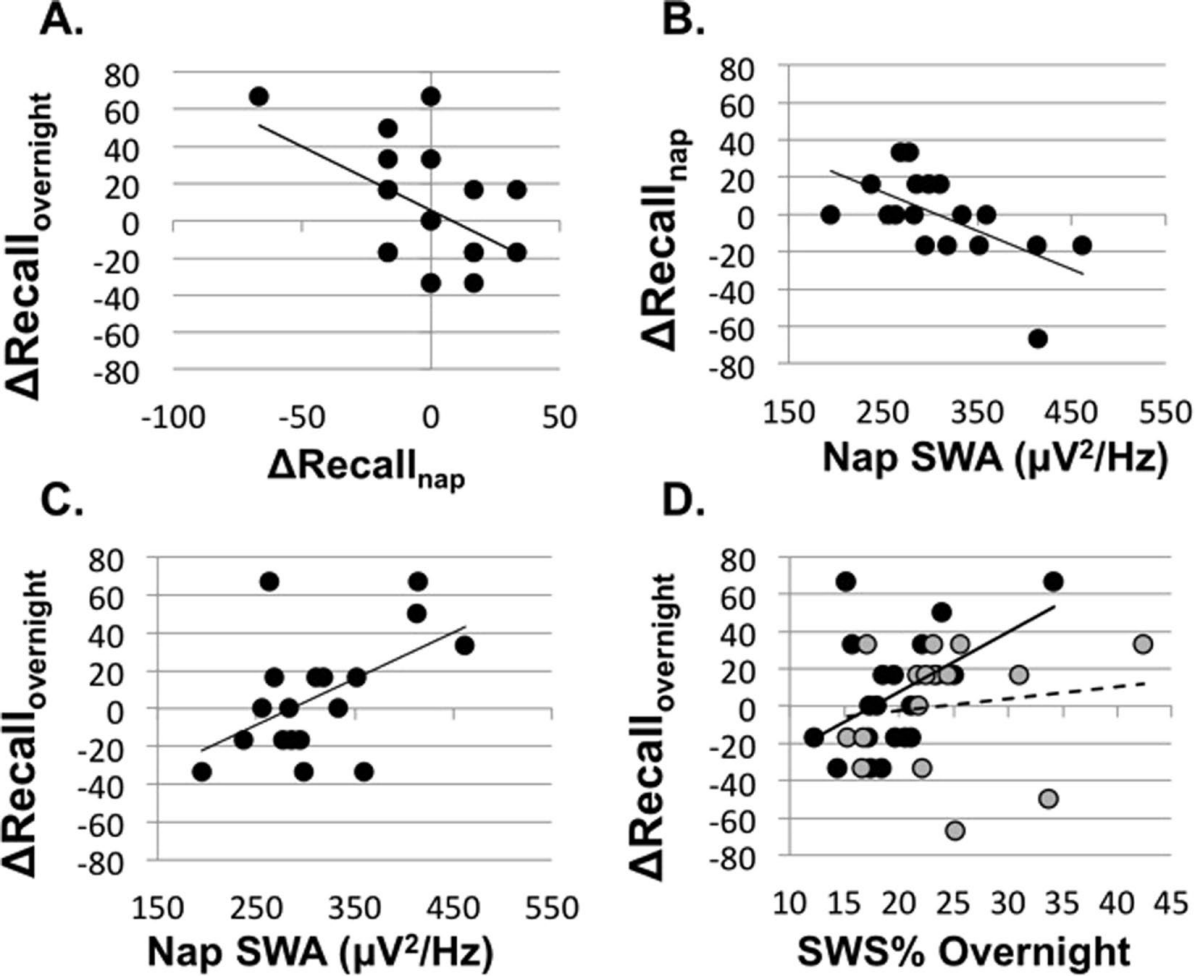
(**A**) Performance decrements across the nap predicted performance improvements overnight (*p* = 0.029). All data points are within 1.5*IQR for both ΔRecall_overnight_ and ΔRecall_nap_. (**B**) SWA in the nap predicted performance decay across the nap (*p* = 0.008). (**C**) Nap SWA also predicts the performance improvements across nocturnal sleep (*p* = 0.028). (**D**) Nocturnal SWS is associated with overnight performance improvements for the nap condition (black/solid line; *p* = 0.029), but not the wake condition (grey/dashed line; *p* = 0.606).

These results suggest a delayed benefit of the nap on emotional memory. We found a similar delayed benefit for procedural learning^12^. In that study, ΔRecall_nap_ predicted ΔRecall_overnight_, suggesting an interaction of multiple sleep bouts on memory processing. Notably, here too with emotional memories, we found a significant association between ΔRecall_nap_ and ΔRecall_overnight_ such that declines in accuracy across the nap were associated with improvements across overnight sleep (*B* = −0.488, *p* = 0.029; 95% CI: [−1.294, −0.079]; Fig. 3A).

Nap habituality was also examined with respect to the nap benefit in post hoc exporatory analyses. Nap habituality was obtained through caregiver report of nap frequency. We did not receive caregiver reports for 9 of the children. Habitually napping children (n = 18) were defined as those who napped 5 or more days per week, whereas nonhabitually napping children (n = 9) were defined as children who napped fewer than 2 days per week (as in^13^). There were no baseline differences (no differences at immediate recall) across conditions for either habitually napping children (*F*(1,17) = 2.236, *p* = 0.153) or nonhabitually napping children (*F*(1,8) = 1.138, *p* = 0.391).

For habitually napping children, performance changes over the nap did not differ from those over wake (ΔRecall_nap/wake_; *F*(1,17) = 0.009, *p* = 0.925). There was also no main effect of emotion (*F*(1,17) = 0.208, *p* = 0.654), nor an emotion by condition interaction (*F*(1,17) = 0.028, *p* = 0.870) across the nap/wake period. However, there was a significant main effect of condition on performance changes across overnight sleep (ΔRecall_overnight_; *F*(1,13) = 4.57, *p* = 0.05, ηp^2^ = 0.260). When habitually napping children had napped during the prior day, they showed protected memory (one-sample t-test; *t*(15) = 1.420, *p* = 0.176; 95% CI: [−5.50, 27.44]) across the overnight sleep bout, whereas significant forgetting was observed for the wake condition (one-sample t-test; *t*(15) = −2.501, *p* = 0.024; 95% CI: [−30.85, −2.46]). Positive and negative stimuli were similarly benefited across overnight sleep (main effect emotion: *F*(1,13) = 0.521, *p* = 0.483; emotion x condition: *F*(1,13) = 0.271, *p* = 0.611). Similarly, for ΔRecall_24-hr_, there was a significant main effect of condition for habitually napping children (*F*(1,13) = 7.581, *p* = 0.016, ηp^2^ = 0.368). Again, the nap was associated with protected memory across the delay (one-sample t-test; *t*(15) = 1.398, *p* = 0.182; 95% CI: [−6.28, 30.21]), whereas the wake condition demonstrated significant forgetting (one-sample t-test; *t*(15) = −3.314, *p* = 0.005; 95% CI: [−35.17, −7.64]). There was no main effect of emotion (*F*(1,13) = 0.223, *p* = 0.645), nor an emotion x condition interaction (*F*(1,13) = 0.290, *p* = 0.599). These results suggest that napping is critical for the retention of memory for habitually napping children. In addition, for habitually napping children, napping in conjunction with overnight sleep equally protects memory for both positively and negatively valenced stimuli.

For nonhabitually napping children, there were no effects of condition (*F*(1,8) = 0.559, *p* = 0.476), nor emotion (main effect emotion: *F*(1,8) = 0.207, *p* = 0.661; emotion x condition: *F*(1,8) = 0.087, *p* = 0.776) on ΔRecall_nap/wake_. Across the overnight sleep bout (ΔRecall_overnight_) however, despite no main effect of condition (*F*(1,8) = 0.105, *p* = 0.755), or emotion (*F*(1,8) = 1.07, *p* = 0.331), there was a significant emotion x condition interaction (*F*(1,8) = 5.274, *p* = 0.05, ηp^2^ = 0.397). Similar trends were observed for ΔRecall_24-hr_ but the interaction was not significant (main effect condition: *F*(1,8) = 0.379, *p* = 0.555; main effect emotion: *F*(1,8) = 0.943, *p* = 0.360; emotion x condition: *F*(1,8) = 4.248, *p* = 0.073).

For both ΔRecall_overnight_ (one-sample t-test; *t*(8) = 2.405, *p* = 0.043; 95% CI: [1.07, 5.12]) and for ΔRecall_24-hr_ (one-sample t-test; *t*(8) = 2.302, *p* = 0.05; 95% CI: [−0.4, 44.49]), napping during the day lead to significantly improved memory for negative stimuli, but not for positive stimuli (one-sample t-tests - ΔRecall_overnight_: *t*(8) = −0.898, *p* = 0.395; ΔRecall_24-hr_: *t*(8) = −1.004, *p* = 0.345). Staying awake during naptime for the nonhabitually napping children did not impact memory for either negative (one-sample t-tests - ΔRecall_overnight_: *t*(8) = −0.241, *p* = 0.815; ΔRecall_24-hr_: *t*(8) = −0.26, *p* = 0.801) or positive stimuli (one-sample t-tests - ΔRecall_overnight_: *t*(8) = 0.546, *p* = 0.600; ΔRecall_24-hr_: *t*(8) = −0.007, *p* = 0.995). These results indicate that while napping is not necessary for nonhabitually napping children to retain memories learned earlier in the day, napping may still help to prioritize and improve memory for salient emotional information.

### Sleep Physiology

Due to issues with lost electrodes (n = 3) or computer malfunction (n = 2), 1 nap record and 4 wake night records were unable to be scored for sleep stages. However, we were able to determine sleep latency on all 4, and REM latency on 2 of the unscorable wake night records, as the recording issues did not occur until later in the night.

Consistent with our prior work^6^, the naps were primarily comprised of SWS and non-REM stage 2 sleep (nREM2; Table 2). Only 6 of the 20 children with PSG reached REM in their nap (*M% REM* = 2.56%, SD = 4.51%). For those 6 children who reached REM sleep during their naps, REM comprised only 8.11% (*SD* = 4.38) of the nap.

**Table 2:** Sleep characteristics from polysomnography (Mean (SD)).

	Nap	Nap Overnight	Wake Overnight	p*
TST (min)	70.88 (24.37)	**522.68 (71.34)**	**549.51 (74.76)**	**0.006**
Sleep Latency (min)	15.03 (10.04)	**44.70 (31.35)**	**26.73 (21.00)**	**0.017**
WASO (min)	11.35 (16.32)	37.08 (40.14)	36.39 (28.22)	0.550
Sleep Efficiency (%)	73.06 (17.78)	**86.54 (7.94)**	**90.03 (6.41)**	**0.033**
nREM1(%)	9.39 (4.49)	**9.39 (2.51)**	**8.42 (2.19)**	**0.004**
nREM2 (%)	36.85 (13.52)	53.22 (4.78)	50.31 (9.05)	0.322
SWS (%)	51.20 (15.36)	**19.73 (4.74)**	**23.89 (7.00)**	**0.003**
REM (%)	2.56 (4.51)	17.67 (3.60)	17.41 (3.96)	0.624
SWA C3 (μV^2^/Hz)	312.07 (67.52)	244.42 (76.96)	243.39 (65.20)	0.970

Note TST = total sleep time; WASO = wake after sleep onset; nREM = non-rapid eye movement sleep, SWS = slow wave sleep; REM = rapid eye movement, SWA = slow wave activity.

*p values correspond to paired samples t-tests comparing overnight bouts across conditions.

We compared overnight sleep from the nap and wake conditions. When children napped, it took them longer to fall asleep at night (*t*(19 = 2.612, *p* = 0.017; 95% CI: [−3.569, 32.381]), they had reduced overnight sleep efficiency (*t*(15) = −2.35, *p* = 0.033; 95% CI: [−9.231, −0.456]), and reduced nocturnal sleep time (*t*(15) = −3.173, *p* = 0.006; 95% CI: [−65.781, −12.919]) compared to the night following the wake condition. Notably, when considering the total daily sleep time (nap + overnight sleep), children had more sleep when they napped (*M* = 579.88, *SD* = 76.18) compared to the wake condition (overnight sleep only; *M* = 549.51, *SD* = 74.75; *t*(15) = 2.25, *p* = 0.04; 95% CI: [1.637, 59.113]).

There were no differences in the percent of the night spent in nREM2 (*t*(15) = 1.03, *p* = 0.322) or REM sleep (*t*(15) = 0.501, *p* = 0.624) across conditions. However, a significantly larger percent of the night was spent in nREM1 (*t*(15) = 3.37, *p* = 0.004; 95% CI: [0.554, 2.458]) and a smaller percent of the night was spent in SWS (*t*(15) = −3.62, *p* = 0.003; 95% CI: [−6.226, −1.611]) in the nap compared to the wake condition. There were no significant differences in overnight SWA across conditions, in either the frontal (*t*(16) = 0.021, *p* = 0.983; 95% CI: [−58.232, 59.422]) or central (*t*(16) = 0.0908, *p* = 0.923; 95% CI: [−53.308, 58.485]) cortical leads.

The presence of REM in the nap did not affect overnight REM quantity (*t*(17) = −1.539, *p* = 0.142) or latency (*t*(17) = −1.456, *p* = 0.164); further, the percentage of the nap spent in REM sleep did not predict the percentage of the night spent in REM sleep (*B* = 0.317, *p* = 0.499). The percentage of the nap spent in SWS also did not predict the percentage of the night spent in SWS (*B* = 0.165, *p* = 0.186). Interestingly, nap SWA positively predicted overnight SWS percentage, although this was not significant (*B* = 0.442, *p* = 0.066).

### Relationship between Sleep Physiology and Memory Performance

We first considered whether REM sleep contributed to the delayed performance enhancements. The percentage of the nap spent in REM sleep did not predict ΔRecall_nap_ (*B* = 0.083, *p* = 0.736). The percentage of the night spent in REM sleep also did not predict ΔRecall_overnight_ (*B* = −0.350, *p* = 0.131). Interestingly, the percentage of REM sleep across all sleep bouts did significantly but negatively predict ΔRecall_24-hr_ (*B* = −0.458, *p* = 0.049; 95% CI: [−0.001, 0.000]). Greater REM percentage across all sleep bouts was associated with greater memory decline across the 24-hour period.

Given the emerging research supporting a role of SWS and SWA in emotional memory consolidation, we next examined the contribution of these sleep traits on the change in memory performance across sleep bouts. The percentage of the nap spent in SWS sleep did not predict ΔRecall_nap_ (*B* = −0.347, *p* = 0.146); however, there was a significant inverse relationship between ΔRecall_nap_ and nap SWA (*B* = −0.601, *p* = 0.008; 95% CI: [−2.982, −0.517]; Fig. 3B) such that greater nap SWA was associated with more memory decay. While this result could indicate poorer consolidation due to greater sleep need or greater sleep inertia of the child, nap SWA was not significantly correlated with sleepiness at either immediate (*r* = −0.014, *p* = 0.955) nor delayed recall (*r* = −0.056, *p* = 0.825). Further, the relationship between SWA and memory decay was driven by reduced memory for positive stimuli (*B* = −0.599, *p* = 0.009; 95% CI: [−2.302, −0.391]) rather than negative stimuli (*B* = −0.422, *p* = 0.081). The percentage of the night spent in SWS sleep did positively predict ΔRecall_overnight_ (*B* = 0.488, *p* = 0.029; 95% CI: [0.008, 0.140]; Fig. 3D). This effect was driven by memory for negative stimuli (*B* = 0.556, *p* = 0.011; 95% CI: [1.713, 11.495]) as compared to positive stimuli (*B* = 0.178, *p* = 0.452; Fig. 3D). Overnight SWA did not predict ΔRecall_overnight_ (*B* = 0.216, *p* = 0.360). The percentage of SWS sleep across all sleep bouts did not significantly predict ΔRecall_24-hr_ (*B* = −0.005, *p* = 0.983), nor was there a significant relationship between ΔRecall_24hr_ and the average SWA across both the nap and the overnight sleep bouts (*B* = −0.081, *p* = 0.936).

In light of the reduced accuracy across the nap predicting improvements in accuracy overnight (Fig. 3A), and the associations between nap SWA and overnight SWS with recognition memory, we investigated possible interactions between these two sleep bouts. Supporting an interaction across sleep bouts, SWA in the nap negatively predicted ΔRecall_nap_ (*B* = −0.601, *p* = 0.008; 95% CI: [−2.982, −0.517]; Fig. 3B) and positively predicted ΔRecall_overnight_ (*B* = 0.518, *p* = 0.028; 95% CI: [0.000, 0.005]; Fig. 3C). Importantly, ΔRecall_overnight_ was also positively predicted by overnight SWS in the nap condition as previously stated; however, this relationship was not observed in the wake condition (*B* = 0.140, *p* = 0.606; Fig. 3D). In other words, overnight SWS predicted improved memory performance when a nap rich in SWA had occurred earlier in the day.

**Figure 4:**
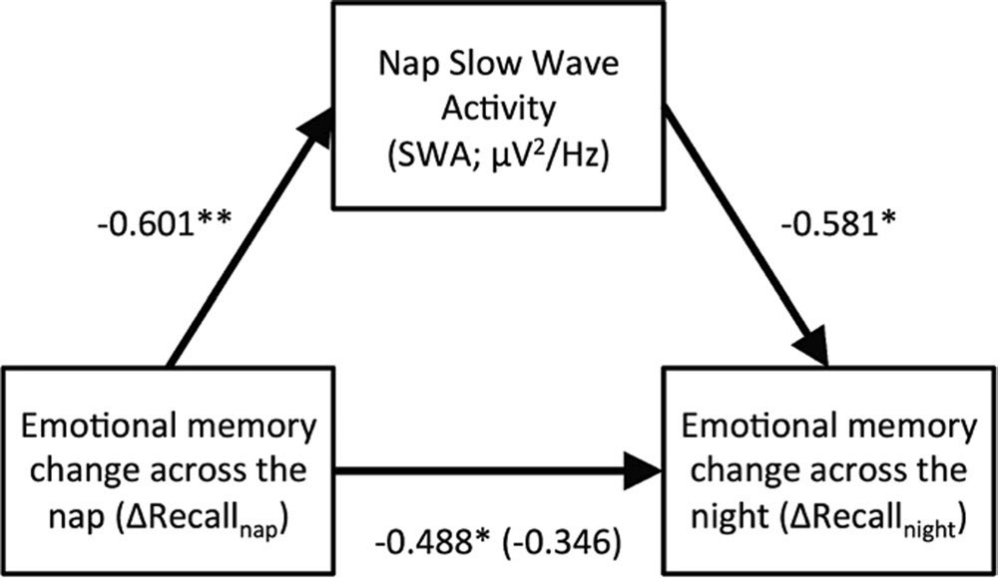
Mediation model of the effect of nap SWA on emotional memory consolidation across both nap and overnight sleep bouts. **p < 0.01, *p < 0.05. Numbers represent the regression coefficient (β). Data indicates full mediation as β becomes non-significant when controlling for the mediator (in parentheses).

Lastly, to further explore the possibility of an interaction between distributed sleep bouts, we assessed whether nap SWA had a mediatory role on memory performance changes. Given that ΔRecall_nap_ significantly predicted ΔRecall_overnight_, and both significantly predicted by nap SWA, the preliminary conditions for a mediation analysis were met. When nap SWA was controlled for, the standardized regression coefficient for the relationship between ΔRecall_nap_ and ΔRecall_overnight_ was reduced from *B* = −0.488, (*p* = 0.029) to *B* = −0.364 (*p* = 0.182). These results support that the relationship between memory performance change across both the nap and overnight sleep bouts was fully mediated by nap SWA (Fig. 4).

## Discussion

Individually, the nap and overnight sleep bouts were not sufficient to induce changes in memory. A significant benefit of napping was observed only when changes across the entire 24-hour period were considered. This supports an interplay between the nap and subsequent overnight sleep in the consolidation of memories in young children. A day of continuous wake led to significant forgetting, particularly for the habitually napping children, which could not be reversed with overnight sleep. The results suggest that the nap led to stabilization and protection of new memories from forgetting even though these effects were not apparent directly following the nap/wake manipulation. In addition, for children who no longer nap regularly, a mid-day nap in conjunction with overnight sleep may still be beneficial for emotional memory organization and prioritization.

The results of this study are consistent with those in procedural memory consolidation in preschool-aged children^13^. Here also, both a nap and subsequent overnight sleep were necessary to observe performance benefits. In that study, preschool children learned a serial reaction time task in the morning. Memory for this procedural task was probed following a nap, and following an equivalent bout of daytime wake (within-subjects design), as well as following the subsequent overnight sleep in both conditions. Memory performance was not significantly different directly following the nap/wake period; however, following overnight sleep, performance was significantly improved when the children had napped the previous day.

A similar delayed benefit of sleep has been demonstrated in juvenile songbirds^14^. Vocal replication of a model song deteriorated when a period of sleep directly followed learning, but this deterioration predicted improvement when assessed long-term. This indicates an active but delayed role of sleep in learning, which may be particularly evident in juvenile populations. The authors of this study concluded that changes across sleep could represent synaptic^15^ or cellular^16^ remodeling, possibly through neuronal replay during sleep. The remodeling across the initial sleep bout presented as performance deterioration but was critical to promote long-term consolidation^15^.

Likewise, SWA in naps in early childhood may yield an initially destabilized memory that is most labile to plastic processes in overnight SWS. While high SWA during the nap was associated with short-term memory decay, it was advantageous to long-term memory consolidation, potentially via the enhancement of overnight SWS. Slow wave activity has often been linked with synaptic remodeling and cortical plasticity^17^. The synchrony of neuronal activation during SWS produces regular and periodic neurotransmitter release as well as changes in intracellular calcium concentrations that facilitate plasticity within neuronal populations^18^. Studies have shown that SWA increases or decreases in cortical regions that are more or less active respectively during previous waking in rats^12^ as well as in humans^19^. Moreover, greater SWA may reflect improved efficiency within the hippocampal-neocortical loop^20^, thereby enhancing memory transfer to long-term storage.

Napping in early childhood may begin the process of synaptic remodeling of the learned memories, which may be sufficient to yield short-term benefits to mood and emotion regulation^10,21^. However, those memories require additional processing over successive sleep periods to promote observable differences in memory performance. Greater plasticity over the nap, as indicated by greater SWA, may allow nocturnal SWS to be more effective at consolidating and preserving the memories learned earlier in the day. In contrast, with the lack of processing and remodeling directly following learning, information is forgotten across the 24-hour period in the wake condition. Newly formed memories may be more susceptible to daytime waking interference^22^ without plastic changes across the nap. This could therefore diminish the likelihood of localized SWA acting to induce plastic changes to stabilize and transfer information into long-term storage during nocturnal sleep.

The results of this study replicate previous findings^6^ that naps in young children contain little-to-no REM sleep. The preschool nap was beneficial to both positive and negative valenced memories despite the lack of REM sleep. In the current study, emotional stimuli were positively associated with nocturnal SWS. These results are consistent with recent reports of SWS being particularly important for emotional memory consolidation in both children^23^ and adults^7,8,20^. Thus, the relative effect of SWS and REM sleep on emotional memory in children is an area that warrants future research.

It is important to note a few limitations to this study. First, the circadian timing of encoding for the laboratory- and classroom-based participants was slightly different (~1.5–2 hours later for the laboratory participants). A second limitation was that there were differences in immediate recognition accuracy across conditions. Change scores were used to assess performance with respect to initial encoding, however the baseline difference could still impact condition effects.

Another limitation was the lack of a neutral stimulus comparison. As such, all stimuli presented to the participants were emotional in valence – either positive or negative. While this design was chosen due to its age-appropriateness and to replicate a previous study using a preschool-aged population^18^, it does hamper direct comparisons to much of the adult emotional memory literature. Further, lacking the neutral stimulus comparison prohibits us from directly assessing the preferential processing of emotional, relative to non-emotional, items as previously reported in young and older adults^24^. Nevertheless, we feel that these results indicate a need for further research into emotional memory consolidation across a nap and subsequent overnight sleep in preschool-aged chidren.

Finally, we failed to replicate the original findings of Kinzler and Shutts^18^ in which memory for negative stimuli was greater than memory for positive stimuli immediately after encoding. While on average the Kinzler and Shutts study demonstrated a negativity bias in emotional recognition memory, they did however report large individual differences in emotional bias (differences in accuracy of recognizing positive or negative stimuli). Of their participants, ~44.7% had a negativity bias in memory, ~42.1% had no emotion bias, and ~13.1% had a positivity bias. In our study, fewer participants showed a lack of emotional bias (28.6%) at immediate recognition, and instead participants were equally as likely to have positive (36.7%) or negative (34.7%) emotional recognition memory bias stimuli. Therefore, the lack of main effect of emotion in our data could reflect a different make up of the participant population, or a generational change in emotional processing of faces presented on a screen.

This study demonstrates that napping is beneficial to memory processing. Given the importance of socio-emotional learning in preschool^25–27^, naps (averaging 70 mins) may support the curricular goals of early childhood education. As such, napping remains an important part of the daily preschool schedule and sufficient time for sleep should be protected.

## Methods

### Participants

Sixty-four children (43 female; *M* = 51.55 months, *SD* = 7.23, range = 34–64 months) participated in the study. Children were required to have normal or corrected-to-normal vision and have no diagnosis (present or past) of sleep or neurological disorders, or learning or developmental disabilities as reported by parents.

### Task

The task was modified from a short-term emotional memory task used by Kinzler and Shutts^1,7,24^ in preschool children. Stimuli were emotionally neutral faces taken from the Radboud Faces Database^28^ and the Max Planck Institute’s FACES collection^29^. An equal number of male and female face stimuli as well as mean and nice statements were presented at encoding. To prevent ceiling effects, the task difficulty was adjusted by age (as in^6^). Older children (>42 months) encoded 18 faces by viewing them for 6 secs each, whereas younger children (<42 months) encoded 12 faces by viewing images for 8 secs each. During the presentation of each image, a statement was read aloud describing the individual as either “mean” or “nice” (e.g., “Jordan is always nice. Today he brought in a book to share with the class.” or “Lena is always nice. Today she helped us pour milk into our cups at lunch time.”). Children were not required to make any responses during encoding. The experimenter visually monitored children to ensure they attended to each stimulus as the statement was read.

In each of three memory assessments (immediate, delayed, and 24-hr recognition), children were given a two-item forced choice recognition task. Children were asked to select the familiar (encoded) face when paired with a novel distractor face, matched for gender. Children who encoded 18 faces were given 6 trials in each of the 3 recognition phases; children who encoded 12 faces were given 4 trials in each recognition phase. Recognition for each encoded image was probed in only one recognition phase. Both the gender and the emotional valence of the targets were balanced across recognition phases.

### Questionnaires

Children self-rated sleepiness using the visual sleepiness scale (VSS^30^) and rated mood using a visual mood scale (VMS^31^. Experimenters also rated the child’s sleepiness and mood on the same scales. Although these measures are not validated for use in this particular age group, the VSS is reliable (compared to Karolinska Sleepiness Scale in adults, α = 0.72^30^; and validated for use in children over 6 yrs^32^. The facial version of the VMS (using faces scaled from happy to sad) has also been shown to be reliable in 5–6 year old children^33^.

Primary caregivers completed the Child Sleep Habits Questionnaire (CSHQ), providing a measure of the child’s sleep habits and sleep health. This assessment is reliable (α = 0.88) and validated for detecting disordered sleep in preschool-aged children (night wakings, parasomnias^34,35^. Consistent with previous work^13^, habitually napping children (n = 18) were defined as children who napped 5 or more days per week, and nonhabitually napping children (n = 9) were defined as children who napped fewer than 2 days per week.

### Procedures

All procedures were approved by the University of Massachusetts Institutional Review Board. Accordingly, procedures were carried out in accordance with the approved protocol. Parents or legal guardians provided written informed consent and child assent was obtained before the testing began. Children participated in two conditions (within-subjects design), a nap and a wake condition, separated by at least 1 week. The order of conditions was counterbalanced across participants. Children were either tested in a preschool classroom (n = 44) or in the sleep laboratory (n = 20) so that sleep physiology could be recorded.

Children tested in the preschool classroom completed the encoding phase and subsequent immediate recognition phase between approximately 10:00 and 11:00 am (Fig. 1). Children then went about their typical classroom routine (e.g., outside playtime, lunch). During the classroom nap opportunity (typically 1:00–3:00 pm), children were either wake or nap promoted. In the wake promotion condition, children were required to remain on their cot/mat in the darkened classroom, but were kept awake as needed with quiet activities (e.g., books, puzzles). In the nap promotion condition, back rubbing and soothing were used to encourage children to sleep as necessary. After the classroom nap opportunity and a short recovery time to overcome sleep inertia (~3:30 pm), the delayed recognition phase took place. Children and experimenters then completed the VSS. The following morning, experimenters returned to the classroom for the 24-hr recognition phase, after which both children and experimenters again completed the VSS. Approximately one week later, the alternate condition (wake or nap promotion) took place with all procedures identical to the first week.

Procedures for children tested in the sleep laboratory were identical to the classroom design with the following exceptions: (1) all children, regardless of age, were given the 18-item image set in order to have more range in performance measures to compare with sleep physiology; (2) to reduce time in the laboratory, encoding and immediate recognition took place between 11:30 am and 12:00 pm; (3) due to the encoding and recognition taking place later, children had only ~1 hour between immediate recall and napping, as opposed to the ~2 hours in the classroom; (4) children and experimenters completed the VMS in addition to the VSS, and these were assessed at the immediate, delayed, and 24-hr recognition phases. Additionally, children who came to the sleep lab were equipped with a PSG montage prior to sleep intervals (Fig. 1). In the nap condition, after PSG application, children were given a 2-hr nap opportunity (~1:00–3:00 pm) in which sleep was promoted. Polysomnography was also applied following immediate recognition in the wake condition to ensure wakefulness. Following the 2-hr nap/wake opportunity, delayed recognition was assessed (~3:30 pm). After delayed recognition, children and parents left the laboratory to complete the rest of their daily routine. In both conditions, parents brought their child back to the laboratory in the evening, approximately 1 hr prior to the child’s typical bedtime, for the PSG montage to be re-applied. In the laboratory, typical bedtime for most children was ~8:00 pm, but ranged from ~7:30–10:00 pm. Children were allowed to sleep as long as they could overnight. Children awoke naturally without influence from the researchers. Most children awoke around 7:00am, although wake times also ranged from ~4:45am–8:00am. In the morning, following removal of the PSG montage and morning routines (e.g., teeth-brushing, changing clothes; approximately 30 mins after waking), 24-hr recognition was tested. It should be noted that the 24-hr recall for the laboratory study was not tested exactly 24-hrs after encoding, given that laboratory encoding occurred ~1.5–2 hours later than encoding classroom encoding.

### Polysomnography

A 32-electrode electroencephalography (EEG) cap (Easy Cap; Brain Products GmbH, Germany), customized with 2 electromyography (EMG) and 2 electrooculography (EOG) electrodes, was used for physiological recordings. The EEG cap included 25 cortical EEG leads (F3, F4, Fz, F7, F8, FCz, FC1, FC2, FC5, FC6, Cz, C3, C4, CP1, CP2, CP5, CP6, Pz, P3, P4, P7, P8, POz, O1, and O2) referenced to electrodes placed on the mastoids (A1 and A2). For two children, the EasyCap was not available and an AURA PSG ambulatory system (Grass Products, Natus Technologies) with a 14-electrode montage including 7 EEG leads, 2 EMG leads, and 2 EOG leads, was used. EEG leads included F3, F4, Cz, C3, C4, O1, and O2 all referenced to electrodes placed on the mastoids (A1 and A2). A ground electrode was affixed to the forehead. EMG electrodes were placed on the chin and referenced to each other.

### Data Analyses

To assess changes in memory over naps and equivalent intervals of wake, baseline recognition accuracy (percent correct) was subtracted from delayed recognition accuracy (ΔRecall_nap/wake_ = delayed recognition − immediate recognition accuracy; Fig. 1). ΔRecall_nap_ was also used for associations with nap physiology and memory performance change across this delay. ΔRecall_overnight_ (24-hr recognition − delayed recognition accuracy) was used to assess the change in memory across the overnight sleep period. ΔRecall_overnight_ was also used to correlate overnight sleep physiology with performance change across the overnight delay. Finally, ΔRecall _24-hr_ (ΔRecall _24-hr_ = 24-hr recognition − immediate recognition accuracy) was calculated to measure the change in performance across the entire 24-hr period.

Effects were assessed with 2 × 2 repeated-measures ANOVAs comparing the difference scores with condition (Nap vs. Wake) and emotion (Mean vs. Nice) as within-subjects factors. To assess the relationship between ΔRecall_nap_ and ΔRecall_overnight_ and between behavior and sleep physiology, linear regressions were used. Paired-samples t-tests were used to examine differences in sleep physiology between the two experimental nights, as well as to assess differences in sleepiness and emotionality.

Lastly, mediation was assessed with the Baron and Kenny method^36^. This method was employed to test the hypothesis that memory performance change across both sleep bouts was mediated by physiological sleep measures. The Baron and Kenny method defines a mediator as a variable that is independently associated with both the predictor and outcome variables, but one that also explains the majority of the variance in the association. As such, the regression coefficient between the predictor and outcome variables must reduce when the mediating variable is controlled for, and for full mediation, the relationship must become non-significant.

PSG recordings were coded for sleep stages using the American Academy of Sleep Medicine criteria^37^, developed for healthy young adults and suitable for young children^6,38^. Additionally, spectral analyses of PSG were run using BrainVision Analyzer 2 software (Ver 2.4; Brain Products GmbH, Germany), with interest in SWA defined with a range of 0.5–4 Hz. Specifically, SWS staged segments were extracted and then analyzed using the periodogram method for spectral analysis^39^. Data were divided into 4-second epochs for artifact rejection on individual channels, and a fast-Fourier transform (FFT) was applied using a Hanning window with 10% overlap and utilizing covariance.

## Data Availability

The data from the current study are available from the corresponding author on reasonable request.
